# Supplementary material for: Hot spot prediction in protein-protein interactions by an ensemble system
Source: BMC Syst Biol. 2018 Dec 31;12(Suppl 9):132. doi: 10.1186/s12918-018-0665-8 (PMC6311905; doi:10.1186/s12918-018-0665-8)
Supplement: Supplementary file 2 — All relevant MATLAB code. A simple Matlab implement of our predictor. (ZIP 19,742 kb) [file 12918_2018_665_MOESM2_ESM.zip › Additional file 2/Read ASA/ASA website/SKEMPI/1JTGA.htm]

 NetSurfP - Protein Surface Accessibility and Secondary Structure Predictions

  

|  |  |  |
| --- | --- | --- |
|  |  | NetSurfP - Protein Surface Accessibility and Secondary Structure Predictions  Technical University of Denmark |

  


---

```
# For publication of results, please cite:
# A generic method for assignment of reliability scores applied to solvent accessibility predictions.
# Bent Petersen, Thomas Nordahl Petersen, Pernille Andersen, Morten Nielsen and Claus Lundegaard
# BMC Structural Biology 2009, 9:51 doi:10.1186/1472-6807-9-51
#
# Column 1: Class assignment - B for buried or E for Exposed - Threshold: 25% exposure, but not based on RSA
# Column 2: Amino acid
# Column 3: Sequence name
# Column 4: Amino acid number
# Column 5: Relative Surface Accessibility - RSA
# Column 6: Absolute Surface Accessibility
# Column 7: Z-fit score for RSA prediction
# Column 8: Probability for Alpha-Helix
# Column 9: Probability for Beta-strand
# Column 10: Probability for Coil
E H  1JTGA                  1    0.845 153.687   0.386   0.000   0.001   0.999
E P  1JTGA                  2    0.504  71.489  -0.749   0.321   0.003   0.675
E E  1JTGA                  3    0.703 122.867   1.400   0.782   0.003   0.216
B T  1JTGA                  4    0.173  24.009   0.381   0.923   0.002   0.076
E L  1JTGA                  5    0.257  47.057   0.777   0.938   0.007   0.055
E V  1JTGA                  6    0.643  98.844   1.320   0.938   0.007   0.055
E K  1JTGA                  7    0.415  85.407   2.238   0.970   0.001   0.030
B V  1JTGA                  8    0.026   4.027   1.014   0.970   0.001   0.030
E K  1JTGA                  9    0.419  86.229   2.447   0.970   0.001   0.030
E D  1JTGA                 10    0.598  86.157   2.197   0.970   0.001   0.030
B A  1JTGA                 11    0.100  11.020   0.862   0.970   0.001   0.030
B E  1JTGA                 12    0.123  21.488  -0.888   0.923   0.002   0.076
E D  1JTGA                 13    0.720 103.709   1.044   0.858   0.002   0.139
E Q  1JTGA                 14    0.705 125.967   0.879   0.694   0.003   0.303
B L  1JTGA                 15    0.167  30.578   0.517   0.176   0.004   0.820
E G  1JTGA                 16    0.733  57.711   0.370   0.005   0.015   0.979
B A  1JTGA                 17    0.132  14.590  -0.085   0.004   0.085   0.910
E R  1JTGA                 18    0.381  87.363   1.472   0.004   0.616   0.381
B V  1JTGA                 19    0.090  13.864   0.940   0.001   0.959   0.040
B G  1JTGA                 20    0.029   2.306   0.108   0.001   0.959   0.040
B Y  1JTGA                 21    0.024   5.150   0.998   0.000   0.983   0.017
B I  1JTGA                 22    0.042   7.696   0.924   0.000   0.983   0.017
B E  1JTGA                 23    0.020   3.546   1.172   0.000   0.983   0.017
B L  1JTGA                 24    0.065  11.828   1.119   0.000   0.983   0.017
B D  1JTGA                 25    0.084  12.148   0.145   0.001   0.900   0.099
B L  1JTGA                 26    0.166  30.358  -0.014   0.004   0.197   0.799
E N  1JTGA                 27    0.652  95.424   0.146   0.004   0.085   0.910
E S  1JTGA                 28    0.427  50.021  -0.151   0.018   0.019   0.964
E G  1JTGA                 29    0.408  32.086  -1.360   0.018   0.019   0.964
E K  1JTGA                 30    0.468  96.350   1.007   0.069   0.386   0.545
E I  1JTGA                 31    0.278  51.338   1.139   0.018   0.846   0.136
B L  1JTGA                 32    0.110  20.086   1.310   0.011   0.918   0.071
B E  1JTGA                 33    0.217  37.927   0.332   0.018   0.846   0.136
B S  1JTGA                 34    0.186  21.752  -0.988   0.085   0.775   0.140
E F  1JTGA                 35    0.341  68.499   0.536   0.074   0.484   0.442
E R  1JTGA                 36    0.296  67.853   0.420   0.018   0.088   0.893
E P  1JTGA                 37    0.472  66.906  -1.330   0.018   0.019   0.964
E E  1JTGA                 38    0.652 113.904  -0.816   0.018   0.019   0.964
E E  1JTGA                 39    0.357  62.298   0.525   0.019   0.141   0.840
E R  1JTGA                 40    0.408  93.432   0.867   0.022   0.359   0.619
B F  1JTGA                 41    0.123  24.626   1.403   0.021   0.279   0.699
B P  1JTGA                 42    0.198  28.025   0.000   0.056   0.142   0.802
B M  1JTGA                 43    0.075  14.987  -1.252   0.257   0.016   0.727
B M  1JTGA                 44    0.027   5.383  -0.587   0.430   0.016   0.555
B S  1JTGA                 45    0.051   5.919  -0.980   0.717   0.014   0.269
B T  1JTGA                 46    0.050   6.949  -0.582   0.923   0.002   0.076
B F  1JTGA                 47    0.050  10.015   0.221   0.923   0.002   0.076
B K  1JTGA                 48    0.053  10.861   0.599   0.970   0.001   0.030
B V  1JTGA                 49    0.043   6.532  -0.052   0.970   0.001   0.030
B L  1JTGA                 50    0.071  12.927   0.208   0.970   0.001   0.030
B L  1JTGA                 51    0.041   7.544   0.086   0.970   0.001   0.030
B C  1JTGA                 52    0.021   2.948   0.847   0.970   0.001   0.030
B G  1JTGA                 53    0.076   5.997   0.258   0.970   0.001   0.030
B A  1JTGA                 54    0.074   8.210   0.839   0.970   0.001   0.030
B V  1JTGA                 55    0.025   3.889   1.027   0.970   0.001   0.030
B L  1JTGA                 56    0.054   9.814   0.949   0.970   0.001   0.030
E S  1JTGA                 57    0.331  38.758   1.371   0.970   0.001   0.030
B R  1JTGA                 58    0.196  44.838   0.479   0.970   0.001   0.030
B I  1JTGA                 59    0.088  16.317   0.180   0.923   0.002   0.076
E D  1JTGA                 60    0.651  93.852   1.062   0.858   0.002   0.139
E A  1JTGA                 61    0.711  78.385   0.939   0.406   0.004   0.590
E G  1JTGA                 62    0.603  47.440  -0.976   0.053   0.005   0.942
E Q  1JTGA                 63    0.706 126.145   0.360   0.016   0.005   0.979
B E  1JTGA                 64    0.143  24.982  -0.157   0.005   0.015   0.979
E Q  1JTGA                 65    0.496  88.657   1.175   0.005   0.015   0.979
B L  1JTGA                 66    0.165  30.266  -0.058   0.018   0.047   0.935
E G  1JTGA                 67    0.619  48.731  -0.241   0.004   0.085   0.910
E R  1JTGA                 68    0.380  86.906   0.426   0.004   0.420   0.576
E R  1JTGA                 69    0.410  93.982   1.289   0.001   0.900   0.099
B I  1JTGA                 70    0.066  12.136   1.003   0.001   0.900   0.099
E H  1JTGA                 71    0.462  83.983   1.595   0.001   0.900   0.099
B Y  1JTGA                 72    0.068  14.574   0.731   0.002   0.816   0.182
E S  1JTGA                 73    0.314  36.801   1.195   0.023   0.655   0.322
E Q  1JTGA                 74    0.587 104.749  -0.056   0.069   0.386   0.545
E N  1JTGA                 75    0.560  81.984   0.034   0.118   0.150   0.732
B D  1JTGA                 76    0.201  28.979  -1.026   0.118   0.150   0.732
E L  1JTGA                 77    0.300  54.857  -0.651   0.125   0.227   0.648
E V  1JTGA                 78    0.462  71.056  -0.816   0.066   0.296   0.638
E E  1JTGA                 79    0.566  98.898  -0.823   0.064   0.216   0.721
E Y  1JTGA                 80    0.564 120.505  -2.357   0.018   0.088   0.893
B S  1JTGA                 81    0.297  34.797  -2.269   0.018   0.047   0.935
B P  1JTGA                 82    0.289  41.009  -2.170   0.019   0.141   0.840
E V  1JTGA                 83    0.323  49.676  -0.459   0.021   0.451   0.528
B T  1JTGA                 84    0.231  32.040  -0.881   0.021   0.451   0.528
E E  1JTGA                 85    0.372  64.971  -1.386   0.021   0.451   0.528
E K  1JTGA                 86    0.299  61.484  -0.438   0.022   0.359   0.619
E H  1JTGA                 87    0.440  80.000   0.182   0.020   0.205   0.775
E L  1JTGA                 88    0.432  79.008  -0.692   0.018   0.088   0.893
E T  1JTGA                 89    0.692  96.050  -1.619   0.018   0.019   0.964
E D  1JTGA                 90    0.485  69.874  -0.145   0.052   0.084   0.864
E G  1JTGA                 91    0.552  43.450   0.764   0.020   0.205   0.775
B M  1JTGA                 92    0.092  18.469   1.397   0.021   0.279   0.699
E T  1JTGA                 93    0.211  29.335   1.281   0.056   0.142   0.802
B V  1JTGA                 94    0.047   7.239   0.765   0.970   0.001   0.030
E R  1JTGA                 95    0.331  75.799   1.450   0.970   0.001   0.030
B E  1JTGA                 96    0.195  33.997   1.140   0.988   0.000   0.012
B L  1JTGA                 97    0.018   3.332   1.500   0.988   0.000   0.012
B C  1JTGA                 98    0.028   3.959   0.778   0.988   0.000   0.012
E S  1JTGA                 99    0.299  35.055   1.503   0.988   0.000   0.012
B A  1JTGA                100    0.078   8.574  -0.047   0.970   0.001   0.030
B A  1JTGA                101    0.028   3.097   0.155   0.970   0.001   0.030
B I  1JTGA                102    0.060  11.174   0.016   0.938   0.007   0.055
B T  1JTGA                103    0.181  25.105   0.481   0.879   0.010   0.111
E M  1JTGA                104    0.270  53.987   0.470   0.622   0.015   0.363
B S  1JTGA                105    0.042   4.887  -1.832   0.257   0.016   0.727
B D  1JTGA                106    0.152  21.903  -1.655   0.176   0.004   0.820
B N  1JTGA                107    0.091  13.366  -0.690   0.782   0.003   0.216
B T  1JTGA                108    0.198  27.435   0.433   0.970   0.001   0.030
B A  1JTGA                109    0.021   2.347   0.920   0.970   0.001   0.030
B A  1JTGA                110    0.019   2.149   1.018   0.970   0.001   0.030
B N  1JTGA                111    0.104  15.240   0.611   0.988   0.000   0.012
B L  1JTGA                112    0.079  14.465   1.361   0.988   0.000   0.012
B L  1JTGA                113    0.023   4.175   1.144   0.988   0.000   0.012
B L  1JTGA                114    0.045   8.148   0.441   0.970   0.001   0.030
E T  1JTGA                115    0.395  54.731   0.670   0.923   0.002   0.076
E T  1JTGA                116    0.282  39.044   0.720   0.858   0.002   0.139
B I  1JTGA                117    0.091  16.890  -0.568   0.502   0.002   0.495
B G  1JTGA                118    0.308  24.255  -1.459   0.053   0.005   0.942
E G  1JTGA                119    0.282  22.225  -1.017   0.016   0.005   0.979
B P  1JTGA                120    0.269  38.100   0.467   0.782   0.003   0.216
E K  1JTGA                121    0.605 124.490   0.205   0.970   0.001   0.030
E E  1JTGA                122    0.336  58.752   1.015   0.975   0.003   0.022
B L  1JTGA                123    0.026   4.834   0.874   0.970   0.001   0.030
B T  1JTGA                124    0.143  19.890   1.337   0.970   0.001   0.030
E A  1JTGA                125    0.527  58.042   1.576   0.988   0.000   0.012
B F  1JTGA                126    0.211  42.448   1.695   0.988   0.000   0.012
B L  1JTGA                127    0.021   3.882   0.720   0.970   0.001   0.030
E H  1JTGA                128    0.488  88.840   1.672   0.970   0.001   0.030
E N  1JTGA                129    0.668  97.810   1.385   0.923   0.002   0.076
B M  1JTGA                130    0.154  30.875   1.215   0.600   0.003   0.397
E G  1JTGA                131    0.507  39.877  -0.813   0.053   0.005   0.942
B D  1JTGA                132    0.111  15.952  -0.176   0.016   0.005   0.979
E H  1JTGA                133    0.595 108.140   0.063   0.018   0.047   0.935
E V  1JTGA                134    0.447  68.704  -0.422   0.020   0.205   0.775
B T  1JTGA                135    0.090  12.511  -0.421   0.021   0.451   0.528
E R  1JTGA                136    0.326  74.631   0.577   0.023   0.655   0.322
B L  1JTGA                137    0.094  17.211  -0.027   0.022   0.552   0.426
E D  1JTGA                138    0.346  49.815  -0.206   0.022   0.359   0.619
E R  1JTGA                139    0.340  77.768  -0.529   0.064   0.216   0.721
E W  1JTGA                140    0.328  78.956  -1.170   0.056   0.142   0.802
E E  1JTGA                141    0.410  71.610  -1.416   0.113   0.087   0.800
E P  1JTGA                142    0.382  54.177  -1.669   0.191   0.086   0.723
E E  1JTGA                143    0.361  63.137  -1.327   0.184   0.043   0.773
B L  1JTGA                144    0.224  41.033  -1.571   0.184   0.043   0.773
B N  1JTGA                145    0.258  37.771  -1.283   0.184   0.043   0.773
E E  1JTGA                146    0.612 106.986  -1.420   0.113   0.043   0.844
E A  1JTGA                147    0.381  41.975  -2.288   0.058   0.017   0.925
E I  1JTGA                148    0.357  66.137  -1.261   0.018   0.019   0.964
E P  1JTGA                149    0.465  65.983  -1.553   0.018   0.019   0.964
E N  1JTGA                150    0.592  86.640  -2.332   0.016   0.005   0.979
E D  1JTGA                151    0.502  72.381  -0.984   0.016   0.005   0.979
E E  1JTGA                152    0.388  67.714  -1.346   0.016   0.005   0.979
E R  1JTGA                153    0.414  94.760   0.189   0.016   0.005   0.979
B D  1JTGA                154    0.174  25.001  -1.580   0.053   0.043   0.903
B T  1JTGA                155    0.137  18.946   0.510   0.056   0.142   0.802
B T  1JTGA                156    0.023   3.135  -0.033   0.052   0.084   0.864
B M  1JTGA                157    0.040   8.024   0.416   0.268   0.043   0.689
B P  1JTGA                158    0.019   2.696   0.877   0.923   0.002   0.076
B V  1JTGA                159    0.188  28.957   1.020   0.970   0.001   0.030
B A  1JTGA                160    0.058   6.392  -0.653   0.970   0.001   0.030
B M  1JTGA                161    0.017   3.382   1.452   0.970   0.001   0.030
B A  1JTGA                162    0.019   2.105   0.838   0.970   0.001   0.030
E T  1JTGA                163    0.203  28.142   1.773   0.970   0.001   0.030
B T  1JTGA                164    0.031   4.286   0.277   0.970   0.001   0.030
B L  1JTGA                165    0.021   3.772   1.144   0.970   0.001   0.030
B R  1JTGA                166    0.171  39.090   0.802   0.988   0.000   0.012
B K  1JTGA                167    0.173  35.648   1.234   0.970   0.001   0.030
B L  1JTGA                168    0.026   4.816   0.576   0.970   0.001   0.030
B L  1JTGA                169    0.125  22.942   0.531   0.923   0.002   0.076
E T  1JTGA                170    0.497  68.878   0.797   0.694   0.003   0.303
E G  1JTGA                171    0.508  40.011   0.301   0.176   0.004   0.820
E E  1JTGA                172    0.657 114.708  -0.059   0.053   0.005   0.942
B L  1JTGA                173    0.267  48.943  -0.635   0.058   0.017   0.925
B L  1JTGA                174    0.075  13.696  -0.265   0.018   0.019   0.964
E T  1JTGA                175    0.437  60.543   1.345   0.018   0.019   0.964
E L  1JTGA                176    0.582 106.491   0.578   0.923   0.002   0.076
E A  1JTGA                177    0.634  69.911   0.531   0.923   0.002   0.076
B S  1JTGA                178    0.111  13.033  -0.888   0.970   0.001   0.030
B R  1JTGA                179    0.134  30.755   0.581   0.970   0.001   0.030
E Q  1JTGA                180    0.560 100.070   0.942   0.988   0.000   0.012
E Q  1JTGA                181    0.274  48.936   1.586   0.970   0.001   0.030
B L  1JTGA                182    0.023   4.156   0.399   0.970   0.001   0.030
B I  1JTGA                183    0.188  34.872   0.712   0.970   0.001   0.030
E D  1JTGA                184    0.511  73.621   0.929   0.970   0.001   0.030
B W  1JTGA                185    0.101  24.387   0.441   0.970   0.001   0.030
B M  1JTGA                186    0.053  10.645  -0.556   0.923   0.002   0.076
E E  1JTGA                187    0.447  78.073   0.325   0.782   0.003   0.216
E A  1JTGA                188    0.610  67.255  -0.957   0.522   0.016   0.462
B K  1JTGA                189    0.224  45.995  -0.625   0.181   0.016   0.803
E V  1JTGA                190    0.392  60.312  -1.086   0.113   0.043   0.844
B A  1JTGA                191    0.317  34.955  -1.318   0.184   0.043   0.773
E G  1JTGA                192    0.335  26.333  -0.761   0.257   0.016   0.727
E P  1JTGA                193    0.521  73.987  -0.323   0.406   0.004   0.590
B L  1JTGA                194    0.180  32.885  -0.013   0.430   0.016   0.555
B L  1JTGA                195    0.038   6.885  -0.289   0.522   0.016   0.462
E R  1JTGA                196    0.287  65.769   0.130   0.455   0.046   0.498
E S  1JTGA                197    0.498  58.319  -1.262   0.339   0.016   0.645
B A  1JTGA                198    0.268  29.556  -1.629   0.257   0.016   0.727
B L  1JTGA                199    0.136  24.993  -0.027   0.115   0.016   0.868
E P  1JTGA                200    0.248  35.205  -0.998   0.058   0.017   0.925
E A  1JTGA                201    0.640  70.583  -1.120   0.058   0.017   0.925
E G  1JTGA                202    0.617  48.558  -0.725   0.018   0.019   0.964
B W  1JTGA                203    0.069  16.570   0.376   0.018   0.088   0.893
E F  1JTGA                204    0.210  42.107   0.067   0.021   0.451   0.528
B I  1JTGA                205    0.060  11.063   0.269   0.021   0.756   0.223
B A  1JTGA                206    0.023   2.590  -0.898   0.023   0.655   0.322
B D  1JTGA                207    0.081  11.701  -0.582   0.022   0.552   0.426
B K  1JTGA                208    0.048   9.874  -0.339   0.022   0.359   0.619
B S  1JTGA                209    0.066   7.782  -0.519   0.021   0.279   0.699
B G  1JTGA                210    0.074   5.808  -1.761   0.021   0.279   0.699
B A  1JTGA                211    0.235  25.842  -0.918   0.056   0.142   0.802
B G  1JTGA                212    0.163  12.804  -0.438   0.113   0.087   0.800
E E  1JTGA                213    0.503  87.944  -0.870   0.113   0.043   0.844
E R  1JTGA                214    0.435  99.592  -0.387   0.115   0.016   0.868
B G  1JTGA                215    0.186  14.654  -1.262   0.113   0.043   0.844
B S  1JTGA                216    0.125  14.603  -0.307   0.064   0.216   0.721
B R  1JTGA                217    0.184  42.136   0.322   0.021   0.451   0.528
B G  1JTGA                218    0.031   2.408  -0.183   0.003   0.718   0.279
B I  1JTGA                219    0.033   6.179   0.452   0.001   0.900   0.099
B I  1JTGA                220    0.021   3.829   0.504   0.001   0.959   0.040
B A  1JTGA                221    0.018   1.995   0.828   0.001   0.959   0.040
B A  1JTGA                222    0.032   3.570   0.193   0.000   0.983   0.017
B L  1JTGA                223    0.025   4.651   0.130   0.001   0.959   0.040
B G  1JTGA                224    0.268  21.115   1.083   0.001   0.959   0.040
E P  1JTGA                225    0.287  40.711   1.135   0.004   0.514   0.481
E D  1JTGA                226    0.797 114.833  -0.002   0.005   0.015   0.979
E G  1JTGA                227    0.817  64.314  -0.676   0.003   0.003   0.994
E K  1JTGA                228    0.411  84.584   0.582   0.005   0.045   0.951
E P  1JTGA                229    0.298  42.300   1.136   0.004   0.514   0.481
B S  1JTGA                230    0.119  13.970   1.399   0.001   0.900   0.099
B R  1JTGA                231    0.031   7.076   1.007   0.001   0.959   0.040
B I  1JTGA                232    0.020   3.681   1.249   0.001   0.959   0.040
B V  1JTGA                233    0.013   1.967   1.733   0.000   0.983   0.017
B V  1JTGA                234    0.013   1.967   1.596   0.001   0.959   0.040
B I  1JTGA                235    0.018   3.256   1.331   0.001   0.959   0.040
B Y  1JTGA                236    0.059  12.630   0.178   0.001   0.959   0.040
B T  1JTGA                237    0.064   8.807  -0.730   0.004   0.514   0.481
E T  1JTGA                238    0.364  50.542  -0.605   0.005   0.045   0.951
E G  1JTGA                239    0.444  34.911  -0.680   0.005   0.015   0.979
E S  1JTGA                240    0.477  55.916  -1.526   0.005   0.015   0.979
E Q  1JTGA                241    0.632 112.839  -0.626   0.016   0.005   0.979
E A  1JTGA                242    0.468  51.618  -1.116   0.053   0.005   0.942
E T  1JTGA                243    0.476  66.077   0.452   0.053   0.005   0.942
E M  1JTGA                244    0.315  63.011   0.007   0.858   0.002   0.139
E D  1JTGA                245    0.654  94.256   1.109   0.923   0.002   0.076
E E  1JTGA                246    0.446  77.951   1.039   0.923   0.002   0.076
B R  1JTGA                247    0.086  19.786  -0.518   0.970   0.001   0.030
B N  1JTGA                248    0.274  40.114   0.291   0.970   0.001   0.030
E R  1JTGA                249    0.524 119.950   2.024   0.970   0.001   0.030
B Q  1JTGA                250    0.110  19.557   1.398   0.970   0.001   0.030
B I  1JTGA                251    0.021   3.922   1.501   0.970   0.001   0.030
E A  1JTGA                252    0.269  29.699   1.476   0.970   0.001   0.030
E E  1JTGA                253    0.474  82.773   1.849   0.970   0.001   0.030
B I  1JTGA                254    0.035   6.531   0.197   0.970   0.001   0.030
B G  1JTGA                255    0.034   2.668   0.112   0.970   0.001   0.030
E A  1JTGA                256    0.405  44.642   1.952   0.988   0.000   0.012
B S  1JTGA                257    0.229  26.792   1.546   0.970   0.001   0.030
B L  1JTGA                258    0.025   4.614   0.648   0.970   0.001   0.030
B I  1JTGA                259    0.142  26.344   0.551   0.923   0.002   0.076
E K  1JTGA                260    0.665 136.873   1.395   0.858   0.002   0.139
E H  1JTGA                261    0.408  74.288   1.251   0.694   0.003   0.303
E W  1JTGA                262    0.340  81.890  -1.659   0.053   0.005   0.942

Explain the output.  Go back.
```

---
